# Nitric oxide versus epoprostenol for refractory hypoxemia in Covid-19

**DOI:** 10.1371/journal.pone.0270646

**Published:** 2022-06-27

**Authors:** Pai B. H. Poonam, Rebecca Koscik, Trong Nguyen, Shefali Rikhi, Hung-Mo Lin

**Affiliations:** 1 Department of Anesthesia, Perioperative and Pain Medicine, Mount Sinai West-Morningside Hospitals, New York, NY, United States of America; 2 Department of Anesthesia, NYU Langone Health, New York, NY, United States of America; 3 Department of Population Health Science and Policy, Mount Sinai West-Morningside Hospitals, New York, NY, United States of America; Royal College of Surgeons in Ireland, IRELAND

## Abstract

**Objective:**

To compare the efficacy and outcomes with inhaled nitric oxide (iNO) and inhaled epoprostenol (iEPO) in patients with refractory hypoxemia due to COVID-19.

**Design:**

Retrospective Cohort Study.

**Setting:**

Single health system multicenter academic teaching hospitals.

**Patients OR subjects:**

Age group of 18–80 years admitted to the medical ICU.

**Interventions:**

Mechanically ventilated patients with COVID-19 infection, who received either iNO or iEPO between March 1^st^, 2020, and June 30^th^, 2020.

**Measurements and main results:**

The primary outcome was the change in the PaO2/FiO2 (P/F) ratio 1 hour after initiation of pulmonary vasodilator therapy. Secondary outcomes include P/F ratios on days 1–3 after initiation, positive response in P/F ratio (increase of at least 20% in PaO2), total days of treatment, rebound hypoxemia (if there was a drop in oxygen saturation after treatment was stopped), ventilator free days (if any patient was extubated), days in ICU, days to extubation, days to tracheostomy, mortality days after intubation, 30-day survival and mortality. 183 patients were excluded, as they received both iNO and iEPO. Of the remaining 103 patients, 62 received iEPO and 41 received iNO. The severity of ARDS was similar in both groups. Change in P/F ratio at one hour was 116 (70.3) with iNO and 107 (57.6) with iEPO (Mean/SD). Twenty-two (53.7%) patients in the iNO group and 25 (40.3%) in the iEPO group were responders to pulmonary vasodilators *n(%)(*p = 0.152) (more than 20% increase in partial pressure of oxygen, Pao2), and 18 (43.9%) and 31 (50%) patients in the iNO and iEPO group (p = 0.685), respectively, had rebound hypoxemia. Only 7 patients in the cohort achieved ventilator free days (3 in the iEPO group and 4 in iNO group).

**Conclusions:**

We found no significant difference between iNO and iEPO in terms of change in P/F ratio, duration of mechanical ventilation, ICU, in-hospital mortality in this cohort of mechanically ventilated patients with COVID-19. Larger, prospective studies are necessary to validate these results.

## Introduction

SARS-CoV-2 is a respiratory pathogen which causes hypoxemia and respiratory damage, often resulting in hospitalization [1]. Huang et al. reports that the clinical symptoms of SARS-CoV-2 include dyspnea, fever, myalgias and/or dry cough [2]. Most patients have a favorable prognosis, only requiring supplemental oxygen with non-invasive measures. However, patients with comorbidities, such as diabetes or obesity, have a higher risk of progressing to acute respiratory distress syndrome (ARDS) or end-organ failure [2]. Wu et al. (2020) performed a retrospective review of 200 SARS-CoV-2 patients and of those patients, 14% developed ARDS [3] with a case fatality rate of 2.3%. The acute respiratory distress syndrome (ARDS) is classified according to the Berlin definition as mild, moderate, and severe based on arterial partial pressure of oxygen (PaO_2_) to fraction of inspired oxygen (FIO_2_) ratio of 300, 200, and 100 mm Hg, respectively. Gattinoni et al proposed two different types of disease pathologies in respiratory failure due to COVID. L-type disease, where there is already loss of hypoxic pulmonary vasoconstriction, is characterized by low elastance, low lung weight and low ventilation to perfusion (V/Q) ratios. This may progress to H-type disease, which resembles “typical ARDS” with low compliance and high lung weight, wherein deranged pulmonary vasoreactivity may result in vasoconstriction, coagulation disorders, microthrombi, and elevated D-dimer levels [4].

Pulmonary vasodilators, such as inhaled epoprostenol (iEPO) and inhaled nitric oxide (iNO), have been used to treat hypoxemia refractory to conventional treatments in patients with ARDS [5, 6]. Titrated doses of iNO and iEPO redistribute blood flow from poorly ventilated shunt areas of the lung to the areas that are well-ventilated with similar efficacy profiles [5].

The inhaled prostaglandins (PGs) epoprostenol (prostaglandin I2 [PGI2]; Flolan) and alprostadil (prostaglandin E1 [PGE1]) decrease intracellular calcium via a cyclic adenosine monophosphate-mechanism causing pulmonary vasodilation. With potential anti-inflammatory and anti-platelet aggregation properties, they might benefit patients with ARDS [7].

Inhalational NO, a selective pulmonary vasodilator originally used in patients with pulmonary hypertension, diffuses into the blood and is rapidly inactivated, hence the vasodilatory effect of iNO is limited largely to the pulmonary circulation. It leads to increased intracellular concentrations of cyclic guanylyl monophosphate (cGMP) as it diffuses across the alveolar-capillary membrane into the subjacent smooth muscle of pulmonary vessels causing smooth muscle relaxation [8] and degradation by hemoglobin (Hb).

Both iEPO and iNO decrease pulmonary vascular resistance and improve oxygenation, however they are associated with the risk of bleeding as they inhibit platelet aggregation. iNO may also cause methemoglobinemia and rebound hypertension whereas iEPO can cause systemic hypotension and tachycardia due to systemic vasodilation [9].

Both iNO and iEPO have been used in mechanically ventilated patients with SARS-CoV-2 infections. However, their efficacy and effects on morbidity and mortality have not been reported [10]. In this study, we aim to ascertain whether there is a clinically significant difference in outcomes between patients receiving iNO and those receiving iEPO.

## Materials and methods

This study is a retrospective analysis of mechanically ventilated patients with laboratory confirmed SARS-CoV-2 infection who were treated with pulmonary vasodilators at a multicenter large academic institution between March 1^st^ 2020 and June 30^th^ 2020. The study was approved by the Mount Sinai Institutional Review Board (Approval number 20–1905). Patients were included if they received either iNO or iEPO during the hospitalization. They were excluded if they didn’t receive either iNO or iEPO, or if they received both treatments. The use of both iNO and iEPO was implemented as standard of care. The choice of pulmonary vasodilator initiation was at the discretion of the treating physician. As this was a retrospective analysis, informed consent was not obtained for this research study. All patients received the same treatment irrespective of involvement in the study.

Based on our institutional protocol, patients were started on pulmonary vasodilator therapy after failing maximal conventional therapy such as recruitment maneuvers, prone positioning, and PEEP more than 15 cmH_2_O. The decision to start a pulmonary vasodilator and any dosing adjustments were at the discretion of the treating attending physician. The dose of iNO ranged from 20 to 80 ppm and that of iEPO ranged from 0.01 to 0.05 mcg/kg/min.

iNO is delivered through a proprietary system, InoMax (Mallinckrodt Pharmaceuticals, Hampton, NJ). Delivery is ensured by a pneumotach device measuring flow rates in the inspiratory limb of the ventilator circuit. The device then inputs the correct amount of NO based on the flow and desired concentration. The dose is titrated to the lowest concentration for positive response, with all dose changes requiring an assessment by a physician. Weaning was attempted in 5 ppm decrements every 4 hours, as tolerated. The FIO_2_ was kept constant during the weaning phase. Heart rate, systemic blood pressure, and oxygen saturations were carefully monitored, and arterial blood gases were obtained every 4–6 hours.

Positive response, for the management of hypoxemia, is defined as a 20% increase in PaO2. An adverse response is defined as a drop below 90 mm Hg for systolic blood pressure, greater than 20% drop in mean arterial pressure (MAP), an increase of greater than 20% in heart rate, a heart rate above 120 or below 60, or any other sign of hemodynamic instability.

The epoprostenol formulation for inhalation (Veletri, Actelion Pharmaceuticals US, and San Francisco, CA) was drawn into a 50 ml syringe and delivered by a continuous nebulizer in line with the inspiratory limb of the ventilator circuit. More specifically, iEPO was delivered via a syringe pump to a Solo nebulizer (Aerogen, Galway, Ireland). It was initiated at 0.01 to 0.05 μg/kg of predicted body weight/minute with stepwise changes in dose based on efficacy and tolerability. Efficacy and tolerability were assessed similar to iNO. Weaning of iEPO was accomplished by titrating down by 0.01 μg/kg of predicted body weight/minute every 2 hours as tolerated.

Demographic data such as age, gender, weight, BMI, ethnicity, history of smoking, comorbidities (hypertension, diabetes, coronary artery disease, pulmonary arterial hypertension, congestive heart failure, asthma, chronic obstructive pulmonary disorder (COPD), obstructive sleep apnea (OSA), cancer, other autoimmune disorders, immunocompromised state) and APACHE Score was recorded (see Table 1). All the medications received during hospital stay, like steroids, tocilizumab, remdesivir, azithromycin, hydroxychloroquine and antifungals were also recorded.

**Table 1:** Baseline characteristics.

Variables		iNO(*n* = 41)	iEPO(*n* = 62)	*p* value
Age Mean(SD)		57.2(12.6)	62.9(10.5)	0.012
Sex	Female	16 (39.0%)	24 (38.7%)	1
	Male	25 (61.0%)	38 (61.3%)	
Weight Mean(SD)		105 (43.8)	87.6 (20.1)	0.0195
BMI Mean(SD)		34.8(9.66)	31.9(6.76)	0.0963
Ethnicity *n (%)* White		10 (24.4%)	8 (12.9%)	0.53

African American		10 (24.4%)	8 (12.9%)	
Hispanic		14 (34.1%)	20 (32.3%)	
Asian		2 (4.9%)	3 (4.8%)	
Others		6 (14.6%)	13 (21.0%)	
Comorbidities				
NIDDM		8 (19.5%)	18 (29.0%)	0.355
IDDM		6 (14.6%)	8 (12.9%)	1
HTN		23 (56.1%)	35 (56.5%)	1
CAD		6 (14.6%)	9 (14.5%)	1
CHF		3 (7.3%)	0 (0%)	0.062
OSA		3 (7.3%)	7 (11.3%)	0.736
COPD		5 (12.2%)	4 (6.5%)	0.53
Asthma		6 (14.6%)	9 (14.5%)	1
PAH		2(4.9%)	0 (0%)	0.311
Active smoker/Vaping		1 (2.4%)	2 (3.2%)	1
Immunocompromised		1(2.4%)	7 (11.3%)	0.139
Cancer		3 (7.3%)	10 (16.1)	0.233
Pressors/Inotrope (Infusion)				
Phenylephrine *n (%)*		13	18	
(Mcg/min)Median(IQR)		105 [50.0–280]	100 [60.0–120]	0.69
Norepinephrine *n (%)*		39	58	
(Mcg/min)Median(IQR)		18.0 [8.00–30.0]	21.4 [10.3–30.0]	0.302
Vasopressin *n (%)*		30	41	
(Units/hour)Median(IQR)		2.40 [1.80–2.40]	2.40 [2.00–2.40]	0.315
Epinephrine n *(%)*		32	11	
(mcg/min)Median(IQR)		11.0 [2.00–30.0]	15.0 [10.5–25.0]	0.702
Sedation (Infusion) *n (%)*				
Fentanyl		22 (53.7%)	22 (35.5%)	0.105
Hydromorphone		31 (75.6%)	51 (82.3%)	0.457
Morphine		1 (2.4%)	7 (11.3%)	0.205
Ketamine		5 (12.2%)	9 (14.5%)	0.966
Midazolam		36 (87.8%)	21 (33.9%)	<0.001
Dexmedetomidine		14 (34.1%)	44 (71.0%)	<0.001
Propofol		32 (78.0%)	54 (87.1%)	0.281
Neuromuscular blocker *n (%)*				
Cisatracurium Infusion		36 (87.8%)	58 (93.5%)	0.478
Concomitant therapy *n (%)*				
Steroids		38 (92.7%)	61 (98.4%)	0.299
Tocilizumab		15 (36.6%)	21 (33.9%)	0.834
Remdesivir		7 (17.1%)	7 (11.3%)	0.558
Hydroxychloroquine		36 (87.8%)	55 (88.7%)	1
Azithromycin		38 (92.7%)	59 (95.2%)	0.924
Antifungals		5 (12.2%)	11 (17.7%)	0.666
Anticoagulation *n (%)*				
Heparin Infusion		23 (56.1%)	48 (77.4%)	0.0383
TPA infusion		10 (24.4%)	17 (27.4%)	0.91
Organ Dysfunction *n (%)*				
Renal Dysfunction		29 (70.7%)	41 (66.1%)	0.784
Liver Dysfunction		16 (39.0%)	18 (29.0%)	0.4
Treatment Modality *n (%)*				
RRT		19 (46.3%)	23 (37.1%)	0.466
ECMO		2 (4.9%)	1 (1.6%)	0.562
Convalescent plasma		11 (26.8%)	11 (17.7%)	0.329
Blood products		10 (24.4%)	7 (11.3%)	0.138
Prone position		27 (65.9%)	54 (87.1%)	0.020

Abbreviations: Hypertension (HTN), coronary artery disease (CAD), non-insulin diabetes mellitus (NIDDM), insulin dependent diabetes mellitus (IDDM), pulmonary artery hypertension (PAH), congestive heart failure (CHF), chronic obstructive pulmonary disease (COPD), obstructive sleep apnea (OSA),tPa-Tissue plasminogen activator, ECMO- extra corporeal membrane oxygenation, RRT- renal replacement therapy.

The primary outcome was change in PaO2/FiO2 (P/F) ratio after 1 hour of vasodilator therapy. Response was termed as positive if there was a change in P/F ratio (more than 20% increase in Pao2). P/F ratio and ventilator mode were recorded daily for the 3 days following initiation of vasodilators. Secondary outcomes included a change in P/F ratio on days 1 to 3 after initiation of therapy, positive response in P/F ratio (more than 20% increase in Pao2), total days of treatment, use of inotropes, sedation, muscle relaxants, plasma therapy, prone positioning, rebound hypoxemia (if there was a drop in oxygen saturation after treatment was stopped), ventilator free days, days in ICU, days to extubation, days to tracheostomy, time-to-death after intubation, 30-day survival and mortality.

### Statistical methods

Descriptive data were reported as number (%), mean (± standard deviation) or median (interquartile range [IQR]). For group comparisons, two-sample t-test or the Wilcoxon-Mann-Whitney test was used for continuous data, and Chi-square or Fisher Exact test was used for categorical data, as appropriate.

To test whether the P/F ratios were the same throughout the perioperative period, a mixed effect model was fit, including time, treatment group, and their interaction as fixed effects. The model was further adjusted for baseline characteristics, comorbidities, and medication covariates that were found to have a p-value<0.2 in the univariate analyses (i.e., age, BMI, pulmonary artery hypertension (PAH), congenital heart failure (CHF), cancer, immunosuppressed status, prone position, fluid products, and heparin), shown in Table 1. An unspecified variance covariate structure was employed for the repeated P/F ratio measurements, as it yielded the smallest Akaike information criterion(AIC) value. As the 30-day survival was only 15 patients, a logistic regression model was used for a secondary analysis as a fit to screen the important protective or risk factors associated with in-hospital mortality in this cohort using the forward selection (entry criterion of 0.1).

Analysis was performed using SAS 9.4 (SAS Institute Inc., Cary, NC). All tests were 2-sided and statistical significance was defined as a p value < 0.05, unless specified.

## Results

A total of 284 patients received pulmonary vasodilators, of which 183 were excluded as they received both iNO and iEPO. A total of 103 patients were analyzed, of which 41 patients received iNO and 62 received iEPO. Baseline demographic data appear in Table 1. The iEPO group was older (62.9 (10.5) vs. 57.2 (12.6) years; p = 0.012) and weighed less (87.6 (20.1) kg vs. 105 (43.8) kg; p = 0.020), compared to the iNO group.

The iEPO group also had a lower prevalence of pulmonary artery hypertension (PAH) (0% vs 4.9%; p = 0.159) and congestive heart failure (CHF) (0% vs. 7.3%; p = 0.062), but a higher prevalence of immunocompromised disease (11.3% vs. 2.4%; p = 0.139) and cancer (16.1% vs 7.3%; p = 0.233) association.

There was also no difference in the number of patients requiring vasopressors and/or inotropes while receiving pulmonary vasodilators between the two groups. The two groups received several and similar concomitant treatments during hospitalization. However, the patients in the iEPO group were more likely to be placed in the prone position (87.1% vs. 65.9%; p = 0.020) and receive heparin treatment (77.4% vs. 56.1%; p = 0.038), but less likely to receive blood products (11.3% vs. 24.4%; p = 0.138).

**Fig 1:**
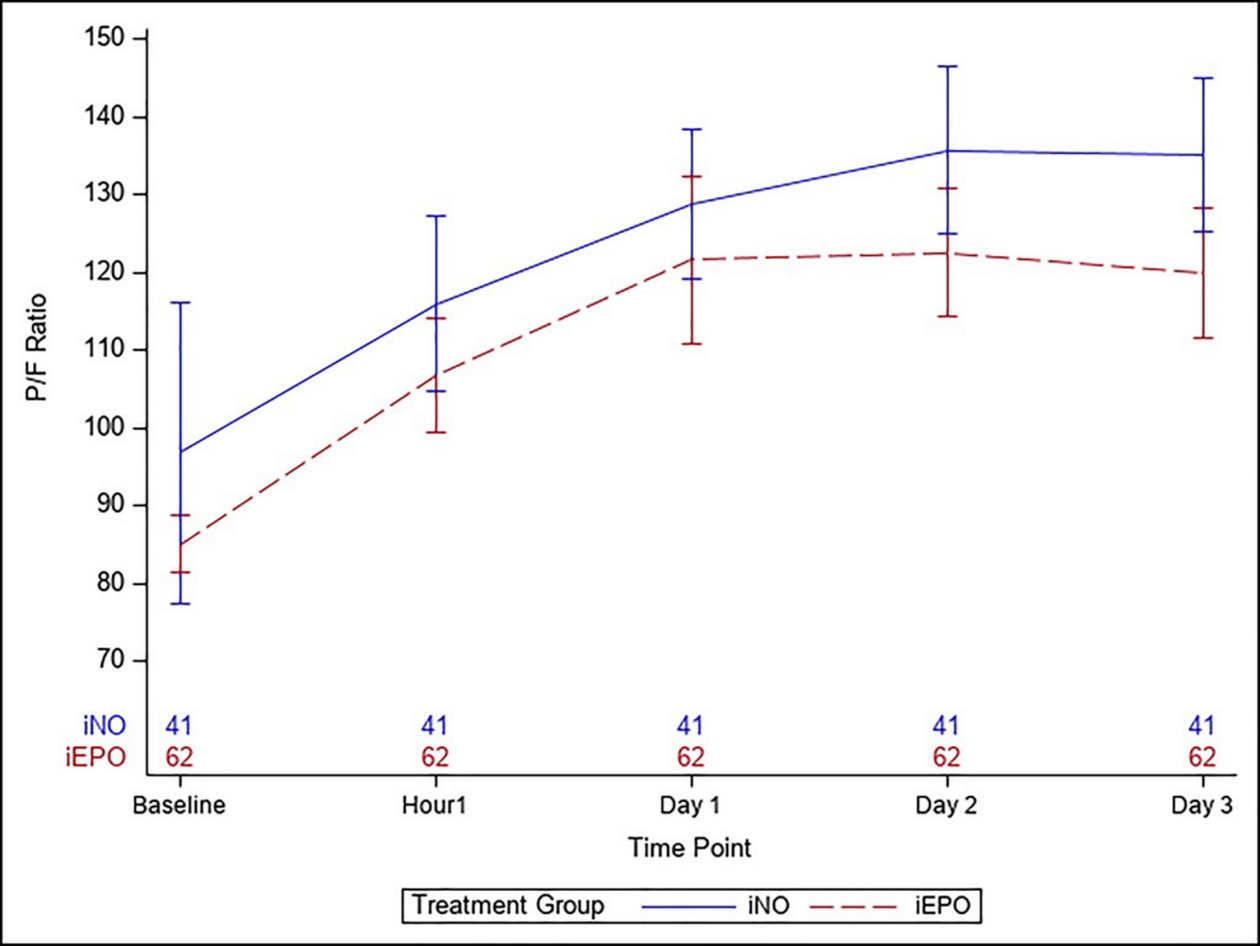
P/F ratio graph. P/F ratio shown baseline (prior to initiation of pulmonary vasodilators), 1 hour after initiation of vasodilator therapy, Day 1, Day 2 and Day 3.

Fig 1 depicts the primary outcome, the P/F ratios, by treatment group at baseline, one hour, and on days 1 to 3, following the onset of the vasodilator therapy. There were no significant differences at baseline (iEPO 85.1 (28.3) vs. iNO 96.8 (124); p = 0.556) as well as the immediate response at one hour (iEPO 107 (57.6) vs. iNO 116 (70.3); p = 0.499). The group trajectories remained similar throughout the entire period, even with the adjustments of the covariates (p = 0.973 for the interaction term between treatment and time). Table 2 describes the details of the respiratory outcomes. There were 14 (23%) patients with moderate ARDS and 48 (77%) patients with severe ARDS in the iEPO group and 9 (22%) patients with moderate ARDS and 32 (78%) patients with severe ARDS in the iNO group. Twenty-two (53.7%) patients in the iNO group and 25 (40.3%) in the iEPO group were termed as responders to pulmonary vasodilators (increase in Pao2 by 20%) (p = 0.152). Eighteen (43.9%) and 31 (50%) in the iNO and iEPO group, respectively, had rebound hypoxemia (p = 0.685).

**Table 2:** Outcomes with use of pulmonary vasodilators.

Variables	iNO(*n* = 41)	iEPO(*n* = 62)	P value
PRE Pao2 Mean(SD)	76.2 (3.04)	76.1 (3.08)	0.946
POST Pa02 Mean(SD)	165 (11.9)	165 (12.1)	0.932
PRE Fio2 Mean(SD)	82.6 (5.71)	82.8 (6.05)	0.825
POST Fi02 Mean(SD)	159 (10.4)	159 (10.8)	0.952
PRE PEEP Mean(SD)	12.4 (3.41)	12.1 (3.35)	0.668
POST PEEP Mean(SD)	24.8 (3.26)	24.5 (3.50)	0.636
Change in PaO2 Mean(SD)	0.931 (0.105)	0.927 (0.108)	0.922
Change in Fi02 *n (n %)*	41 (100%)	62 (100%)	0.847
Change in PEEP *n (n %)*	41 (100%)	62 (100%)	0.864
TV Mean(SD)	432 (34.3)	433 (36.8)	0.871
RR Mean(SD)	22.7 (3.27)	22.6 (3.39)	0.879
PIP Mean(SD)	34.2 (3.60)	34.5 (3.48)	0.663
MAP Mean(SD)	20.9 (2.45)	20.9 (2.48)	0.908
Baseline P/F Ratio Mean(SD)	96.8 (124)	85.1 (28.3)	0.556
Baseline Ventilator Mode			0.0119
AC	15 (36.5%)	40 (64.5%)	
PC	1 (2.4%)	0 (0%)	
PRVC	20 (48.8%)	12 (19.4%)	
PS	4 (9.8%)	7 (11.3%)	
VC	1 (2.4%)	3 (4.8%)	
Baseline Fluid Balance Mean(SD)	411 (1020)	579 (1150)	0.452
Immediate Positive Response Mean(SD)	22 (53.7%)	25 (40.3%)	0.17
P/F Ratio After 1 Hour Mean(SD)	116 (70.3)	107 (57.5)	0.499
Immediate Ventilator Mode Mean(SD)			0.0637
AC	15 (36.5%)	40 (64.5%)
PC	1 (2.4%)	0 (0%)
PRVC	18 (43.9%)	14 (22.6%)
PS	4 (9.8%)	6 (9.7%)
VC	3(7.2%)	2 (3.2%)
P/F Ratio Day 1 Mean(SD)	129 (60.0)	122 (81.6)	0.616
Positive Response Day 1 Mean(SD)	29 (70.7%)	28 (45.2%)	0.0378
Fluid Balance Day 1 Mean(SD)	1010 (1840)	716 (1090)	0.391
Ventilator Mode Day 1 Mean(SD)	NC	NC	
P/F Ratio Day 2 Mean(SD)	136 (61.7)	123 (58.4)	0.339
Positive Response Day 2 Mean(SD)	24 (58.5%)	24 (38.7%)	0.0589
Ventilator Mode Day 2 Mean(SD)	NC	NC	
P/F Ratio Day 3 Mean(SD)	135 (54.2)	120 (53.6)	0.245
Positive Response Day 3 Mean(SD)	22 (53.7%)	20 (32.3%)	0.0476
Ventilator mode Day 3 Mean(SD)	NC	NC	

Abbreviations: Pao2- partial pressure oxygen, Fi02- fractional inspired oxygen concentration, PEEP- positive end

expiratory pressure, TV- tidal volume, RR- respiratory rate, PIP- Peak inspiratory pressure, MAP- mean airway

pressure, P/F ratio- Pao2/Fio2 ratio, AC- assist control, VC- volume control, PC- pressure control, PRVC- pressure

regulated volume control, PS- pressure support, NC- No change.Fio2 POST = Fio2+change in Fio2. Pao2

POST = Pao2+Change in Pao2, PEEP POST = PEEP+ Change in PEEP.

The median total days of treatment was 4 [IQR 2–6] days in the iNO group and 4 [2–5] days in the iEPO group (p = 0.565). The median days to extubation was 21.5 [16.3–25.8] in the iNO group and 11.0 [8.8–13.8] in the iEPO group (p = 0.062). The number of days spent in ICU was also similar (iNO 15 [7–22] vs. iEPO 11 [8, 18]; p = 0.319). The incidence of mortality was

32 (70.1%) and 56 (90.3%) in the iNO and iEPO groups, respectively (p = 0.149). Although the mortality rate appeared to be higher in the iEPO group, the group was also older. The two factors found to be significantly associated with mortality were age (odds ratio 1.07 (95% CI: 1.01 ~ 1.14); p = 0.025) and remdesivir treatment (OR = 0.22 (0.05 ~0.99); p = 0.049). Only seven patients in the cohort achieved ventilator free days (Table 3). There was no difference in the incidence of tracheostomy between the two groups.

**Table 3:** Secondary endpoints.

Variables	iNO(*n* = 62)	iEPO(*n* = 62)	P value
Day of vasodilator therapy initiation after intubation Median (IQR)d	3(1–8)	5(1–9.75)	0.348
APACHE Score Median (IQR)	29.0 [26.0–36.0]	32.5 [27.0–37.3]	0.116
Total days of vasodilator treatment Median (IQR)d	4(2–6)	4 (2–5)	0.565
Rebound hypoxemia SD(Mean)	18 (43.9%)	31 (50.0%)	0.685
Ventilator free days *n (*%)	4(9.8%)	3(4.8%)	0.545
Days in ICU Median (IQR)d	15 (7–22)	11 (8–18)	0.319
Days to extubation Median (IQR)d	21.5 (16.3–25.8)	11 (8.75–13.8)	0.0617
Days to tracheostomy Median (IQR)d	22 (11.8–31.8)	13 (9.5–16.5)	0.157
30-day Survival *n (*%)	12(29.3%)	8(12.9%)	0.0495
Mortality *n (*%)	32(78%)	56(90.3%)	0.149
Terminal extubation *n (*%)	18(43.9%)	23(37.1%)	0.628
Day of mortality after intubation Median (IQR)d	14 (4–19)	10 (7–18)	0.516

Abbreviations: APACHE Score—Acute physiologic assessment and chronic health evaluation score.

## Discussion

This retrospective study conducted in mechanically ventilated patients with COVID-19 evaluated the efficacy of iEPO and iNO for refractory hypoxemia. 70.7% and 45.2% of patients in the iNO and iEPO groups, respectively, had a positive response, defined at an increase of at least 20% in the PaO2. Although the P/F ratio improved in both groups, the difference was not statistically significant.

ARDS is characterized by extensive alveolar damage resulting in leaky alveolar capillaries, and protein-rich pulmonary edema leading to ventilation-perfusion mismatches and hypoxemia [11]. There is a marked maldistribution of pulmonary perfusion in favor of non ventilated, atelectatic areas of the lungs, which is the main cause of pulmonary right-to-left shunting and hypoxemia. Clinical studies in ARDS have demonstrated that the combination of iNO with other interventions, such as high positive end-expiratory pressure (PEEP) and prone positioning, yielded beneficial effects on arterial oxygenation. Aerosolized pulmonary vasodilators such as inhaled iEPO and iNO reduce severe hypoxemia by reducing ventilation-perfusion mismatch, without inducing systemic hypotension, hence have been described as rescue treatments for ARDS [12].

Fullers et al. conducted a meta-analysis of 25 studies which looked at the use of vasodilators in ARDS and concluded that inhaled prostaglandins likely do not improve oxygenation, and that observed effects are secondary to a change in FiO2 or other concomitant therapies like prone positioning or increased positive end-expiratory pressure. In addition, several studies reported that a significant percentage of patients were non-responders to vasodilators. As such, they established that further RCTs are required to demonstrate the benefit with use of PGs in ARDS [7]. Ammar et al. showed that iEPO was non-inferior to iNO for ARDS, with respect to oxygenation and ventilator free days [13].

There is paucity of evidence when it comes to their use in patients with COVID-19. DeGrado et al recently published a study comparing the efficacy of iEPO and iNO in patients with COVID-19. Though they found an improvement in terms of oxygenation and immediate P/F ratio, the difference was insignificant due to a small sample size. Also, there were several interventions including prone positioning, use of tocilizumab, remdesivir, plasma therapy, azithromycin, hydroxychloroquine, steroids and concomitant use of vasoactive agents or neuromuscular agents which might have affected the outcome despite statistical adjustment [14].

Alhazzani et al. published guidelines for management of patients with COVID-19 and recommended against the routine use of iNO in mechanically ventilated adults. A trial of inhaled nitric oxide as a “rescue” therapy, after trying other options, was recommended. They also recommended tapering it to avoid rebound hypoxemia if no good response was seen. This recommendation was based on a Cochrane review of 13 RCTs (1243 patients) of iNO in ARDS, which concluded that there was no significant effect on mortality and an increased risk of acute kidney injury. Improvement in oxygenation was transient and not present beyond 24 hours [15]. With respect to inhaled prostaglandins, there remains a paucity of adequately powered RCTs to recommend their use for severe ARDS [16].

Lotz et al. conducted a retrospective observational study in five patients where pulmonary vasoreactivity, pulmonary shunt fraction and arterial oxygenation was measured via pulmonary arterial catheter 15–30 minutes after initiation of iNO and found that there was improvement in PaO2 and minimal pulmonary vasodilation with no change in shunt fraction. Considering the proposed L- and H-phenotypes of ARDS in COVID-19, they recommend starting iNO therapy ideally in the early transition between the two types, when there is increased shunting, when recruit ability is not lost [17].

Li et al. conducted a study on patients with COVID-19 and refractory hypoxemia and concluded that the combined use of iEPO and proning improved oxygenation, including some patients who did not respond to either proning or iEPO individually [17, 18]. However, they also opined that it is difficult to predict responsiveness because COVID-19 causes sepsis-related endothelial dysfunction, and dysregulation of both nitric oxide and prostacyclin mediated signaling, leading to pulmonary and systemic endothelial dysfunction [19–21].

There are several limitations to our study. Firstly, we were not able to determine post-treatment static compliance or number of hours of proning based on retrospective chart review. Even though the baseline variables were adjusted, it may not adequately account for all the baseline differences between patients who received iEPO and iNO. This was evident by the difference in baseline PaO_2_/FiO_2_ ratio between the two groups which however was not significant. In addition, there are noticeable numeric differences between the two groups in therapeutic interventions before and after initiation of inhaled therapy which could not be controlled statistically. Overall, there were no discernible trends where one group of patients distinctively received more conventional or rescue therapies with the exception of proning. Hence, we believe these differences in the management of ARDS were most likely associated with variations in clinical practice among critical care and anesthesia providers at different hospital sites. Due to the retrospective nature of this study, the preference behind the vasodilator choice and the rationale behind choosing one over the other is hard to decipher. We also excluded the subset of patients who received both to avoid confounding bias. Differences in dosing protocols between sites may also limit the clinical applicability of our study as it is unclear whether findings from the current study may be generalized to other dosing protocols. In addition, the mortality rate observed in this study was significantly higher than that reported by Degrado et al [14]. As our ventilator parameters were consistent with guidelines, the high mortality is likely a reflection of the extreme severity of illness in this subset of COVID -19 patients treated with inhaled vasodilators.

## Conclusions

We compared efficacy and outcomes with iNO and iEPO in mechanically ventilated patients with COVID-19. We found no difference in change in P/F ratio, response rate, duration of mechanical ventilation, ICU stay, in-hospital mortality, or rate of tracheostomy. However, larger prospective trials might be necessary to validate these results.

## Supporting information

S1 File (DOCX)
