# Supplementary material for: Nitric oxide versus epoprostenol for refractory hypoxemia in Covid-19
Source: PLoS One. 2022 Jun 27;17(6):e0270646. doi: 10.1371/journal.pone.0270646 (PMC9236233; doi:10.1371/journal.pone.0270646)
Supplement: S1 File — (DOCX) [file pone.0270646.s001.docx]

####################################################

# Title: Covid gas#

# Author: Yuxia Ouyang #

# Start Date: 06.2021 #

####################################################

# Study Object:

# 1) Group comparison by Treat (NO/EPO)

| Descriptive Summary by Treat | | | | |
| --- | --- | --- | --- | --- |
|  | **NO (N=41)** | **EPO (N=62)** | **Mean & Median Comparison P-value** | **Total (N=103)** |
| **Age** |  |  |  |  |
| Mean (SD) | 57.2 (12.6) | 62.9 (10.5) | T test p=0.0191 | 60.6 (11.7) |
| Median [Q1, Q3] | 58.0 [51.0, 64.0] | 64.0 [56.3, 70.0] | KW test p=0.0118 | 61.0 [53.5, 69.0] |
| **BMI** |  |  |  |  |
| Mean (SD) | 34.8 (9.66) | 31.9 (6.76) | T test p=0.0963 | 33.1 (8.15) |
| Median [Q1, Q3] | 32.7 [27.7, 41.4] | 30.0 [27.4, 36.3] | KW test p=0.133 | 31.2 [27.4, 38.8] |
| Missing | 0 (0%) | 2 (3.2%) |  | 2 (1.9%) |
| **Weight** |  |  |  |  |
| Mean (SD) | 105 (43.8) | 87.6 (20.1) | T test p=0.0195 | 94.6 (32.6) |
| Median [Q1, Q3] | 96.3 [72.6, 117] | 85.6 [77.0, 97.3] | KW test p=0.0319 | 90.5 [73.9, 100] |
| Missing | 1 (2.4%) | 0 (0%) |  | 1 (1.0%) |
| **Phenylephrine gtt** |  |  |  |  |
| Mean (SD) | 152 (119) | 116 (85.8) | T test p=0.371 | 132 (101) |
| Median [Q1, Q3] | 105 [50.0, 280] | 100 [60.0, 120] | KW test p=0.69 | 100 [55.5, 160] |
| Missing | 28 (68.3%) | 45 (72.6%) |  | 73 (70.9%) |
| **Norephinephrine gtt** |  |  |  |  |
| Mean (SD) | 27.1 (46.5) | 25.4 (17.0) | T test p=0.83 | 26.1 (32.1) |
| Median [Q1, Q3] | 18.0 [8.00, 30.0] | 21.4 [10.3, 30.0] | KW test p=0.302 | 20.0 [10.0, 30.0] |
| Missing | 2 (4.9%) | 4 (6.5%) |  | 6 (5.8%) |
| **Vasopressin gtt** |  |  |  |  |
| Mean (SD) | 2.45 (0.942) | 2.53 (0.831) | T test p=0.726 | 2.50 (0.874) |
| Median [Q1, Q3] | 2.40 [1.80, 2.40] | 2.40 [2.00, 2.40] | KW test p=0.315 | 2.40 [1.80, 2.40] |
| Missing | 11 (26.8%) | 21 (33.9%) |  | 32 (31.1%) |
| **Epinephrine gtt** |  |  |  |  |
| Mean (SD) | 48.9 (97.1) | 16.5 (10.1) | T test p=0.347 | 31.1 (65.5) |
| Median [Q1, Q3] | 11.0 [2.00, 30.0] | 15.0 [10.5, 25.0] | KW test p=0.702 | 14.6 [5.00, 30.0] |
| Missing | 32 (78.0%) | 51 (82.3%) |  | 83 (80.6%) |
| **Day after Intubation** |  |  |  |  |
| Mean (SD) | 5.00 (5.42) | 6.27 (6.43) | T test p=0.281 | 5.77 (6.05) |
| Median [Q1, Q3] | 3.00 [1.00, 8.00] | 5.00 [1.00, 9.75] | KW test p=0.348 | 4.00 [1.00, 9.00] |
| **Initial PaO2: FiO2 ratio** |  |  |  |  |
| Mean (SD) | 96.8 (124) | 85.1 (28.3) | T test p=0.556 | 89.8 (81.1) |
| Median [Q1, Q3] | 68.0 [58.0, 94.4] | 81.3 [63.2, 95.0] | KW test p=0.167 | 77.8 [61.0, 95.0] |
| **Initial Fluid Balance** |  |  |  |  |
| Mean (SD) | 411 (1020) | 579 (1150) | T test p=0.452 | 515 (1100) |
| Median [Q1, Q3] | 154 [-60.0, 716] | 425 [1.30, 1180] | KW test p=0.339 | 335 [-3.08, 1000] |
| Missing | 4 (9.8%) | 1 (1.6%) |  | 5 (4.9%) |
| **Initial Apache** |  |  |  |  |
| Mean (SD) | 30.2 (6.71) | 32.4 (6.76) | T test p=0.103 | 31.5 (6.80) |
| Median [Q1, Q3] | 29.0 [26.0, 36.0] | 32.5 [27.0, 37.3] | KW test p=0.116 | 31.0 [27.0, 37.0] |
| Missing | 0 (0%) | 2 (3.2%) |  | 2 (1.9%) |
| **Immediate P:F ratio** |  |  |  |  |
| Mean (SD) | 116 (70.3) | 107 (57.5) | T test p=0.499 | 110 (62.5) |
| Median [Q1, Q3] | 95.0 [72.0, 133] | 94.3 [72.9, 116] | KW test p=0.608 | 95.0 [72.5, 133] |
| Missing | 2 (4.9%) | 0 (0%) |  | 2 (1.9%) |
| **P:F ratio, Day 1** |  |  |  |  |
| Mean (SD) | 129 (60.0) | 122 (81.6) | T test p=0.616 | 125 (73.3) |
| Median [Q1, Q3] | 129 [78.9, 172] | 98.0 [82.0, 146] | KW test p=0.239 | 106 [79.8, 157] |
| Missing | 2 (4.9%) | 5 (8.1%) |  | 7 (6.8%) |
| **Fluid Balance 24h, Day 1** |  |  |  |  |
| Mean (SD) | 1010 (1840) | 716 (1090) | T test p=0.391 | 832 (1430) |
| Median [Q1, Q3] | 809 [156, 1410] | 507 [18.7, 1210] | KW test p=0.487 | 523 [47.3, 1290] |
| Missing | 6 (14.6%) | 7 (11.3%) |  | 13 (12.6%) |
| **P:F ratio, Day 2** |  |  |  |  |
| Mean (SD) | 136 (61.7) | 123 (58.4) | T test p=0.339 | 128 (59.7) |
| Median [Q1, Q3] | 136 [94.3, 158] | 106 [84.0, 160] | KW test p=0.247 | 121 [86.0, 160] |
| Missing | 8 (19.5%) | 13 (21.0%) |  | 21 (20.4%) |
| **P:F ratio, Day 3** |  |  |  |  |
| Mean (SD) | 135 (54.2) | 120 (53.6) | T test p=0.245 | 126 (54.0) |
| Median [Q1, Q3] | 129 [108, 167] | 106 [91.6, 140] | KW test p=0.135 | 116 [91.8, 154] |
| Missing | 11 (26.8%) | 21 (33.9%) |  | 32 (31.1%) |
| **Total days of Tx** |  |  |  |  |
| Mean (SD) | 5.02 (4.30) | 4.29 (2.96) | T test p=0.344 | 4.58 (3.55) |
| Median [Q1, Q3] | 4.00 [2.00, 6.00] | 4.00 [2.00, 5.00] | KW test p=0.565 | 4.00 [2.00, 6.00] |
| **Days in ICU** |  |  |  |  |
| Mean (SD) | 19.0 (22.0) | 14.6 (11.6) | T test p=0.235 | 16.4 (16.6) |
| Median [Q1, Q3] | 15.0 [7.00, 22.0] | 11.0 [8.00, 18.0] | KW test p=0.319 | 12.5 [7.00, 20.8] |
| Missing | 0 (0%) | 1 (1.6%) |  | 1 (1.0%) |
| **Days to Extubation** |  |  |  |  |
| Mean (SD) | 20.6 (8.73) | 11.5 (3.70) | T test p=0.0297 | 17.6 (8.51) |
| Median [Q1, Q3] | 21.5 [16.3, 25.8] | 11.0 [8.75, 13.8] | KW test p=0.0617 | 16.5 [12.0, 23.5] |
| Missing | 33 (80.5%) | 58 (93.5%) |  | 91 (88.3%) |
| **Days to Tracheostomy** |  |  |  |  |
| Mean (SD) | 22.5 (12.3) | 14.1 (8.28) | T test p=0.114 | 19.1 (11.3) |
| Median [Q1, Q3] | 22.0 [11.8, 31.8] | 13.0 [9.50, 16.5] | KW test p=0.157 | 16.0 [10.0, 28.0] |
| Missing | 31 (75.6%) | 55 (88.7%) |  | 86 (83.5%) |
| **Mortality Days after intubation** |  |  |  |  |
| Mean (SD) | 15.1 (13.1) | 13.5 (12.6) | T test p=0.592 | 14.0 (12.8) |
| Median [Q1, Q3] | 14.0 [4.00, 19.0] | 10.0 [7.00, 18.0] | KW test p=0.516 | 11.0 [6.00, 18.0] |
| Missing | 12 (29.3%) | 5 (8.1%) |  | 17 (16.5%) |
| **Pa02** |  |  |  |  |
| Mean (SD) | 76.2 (3.04) | 76.1 (3.08) | T test p=0.946 | 76.1 (3.05) |
| Median [Q1, Q3] | 78.0 [76.0, 78.0] | 77.0 [76.0, 78.0] | KW test p=0.997 | 78.0 [76.0, 78.0] |
| **Fi02** |  |  |  |  |
| Mean (SD) | 82.6 (5.71) | 82.8 (6.05) | T test p=0.825 | 82.7 (5.89) |
| Median [Q1, Q3] | 80.0 [80.0, 90.0] | 80.0 [80.0, 90.0] | KW test p=0.91 | 80.0 [80.0, 90.0] |
| **sp02** |  |  |  |  |
| Mean (SD) | 93.9 (1.69) | 94.0 (1.74) | T test p=0.795 | 93.9 (1.71) |
| Median [Q1, Q3] | 94.0 [92.0, 94.0] | 94.0 [92.0, 94.0] | KW test p=0.819 | 94.0 [92.0, 94.0] |
| **PEEP** |  |  |  |  |
| Mean (SD) | 12.4 (3.41) | 12.1 (3.35) | T test p=0.668 | 12.2 (3.36) |
| Median [Q1, Q3] | 10.0 [10.0, 16.0] | 10.0 [10.0, 16.0] | KW test p=0.673 | 10.0 [10.0, 16.0] |
| **TV** |  |  |  |  |
| Mean (SD) | 432 (34.3) | 433 (36.8) | T test p=0.871 | 432 (35.6) |
| Median [Q1, Q3] | 450 [400, 450] | 425 [400, 450] | KW test p=0.921 | 450 [400, 450] |
| **RR** |  |  |  |  |
| Mean (SD) | 22.7 (3.27) | 22.6 (3.39) | T test p=0.879 | 22.6 (3.33) |
| Median [Q1, Q3] | 20.0 [20.0, 26.0] | 20.0 [20.0, 26.0] | KW test p=0.798 | 20.0 [20.0, 26.0] |
| **PIP** |  |  |  |  |
| Mean (SD) | 34.2 (3.60) | 34.5 (3.48) | T test p=0.663 | 34.4 (3.51) |
| Median [Q1, Q3] | 35.0 [32.0, 35.0] | 35.0 [32.0, 35.0] | KW test p=0.553 | 35.0 [32.0, 35.0] |
| **MAP/PAP** |  |  |  |  |
| Mean (SD) | 20.9 (2.45) | 20.9 (2.48) | T test p=0.908 | 20.9 (2.45) |
| Median [Q1, Q3] | 20.0 [19.0, 21.0] | 20.0 [19.0, 21.0] | KW test p=0.902 | 20.0 [19.0, 21.0] |
| **CHANGE IN PA02** |  |  |  |  |
| Mean (SD) | 88.6 (9.03) | 88.4 (9.12) | T test p=0.928 | 88.5 (9.04) |
| Median [Q1, Q3] | 92.0 [92.0, 93.0] | 92.0 [92.0, 93.0] | KW test p=0.856 | 92.0 [92.0, 93.0] |
| **CHANGE IN PEEP** |  |  |  |  |
| Mean (SD) | 12.4 (3.29) | 12.4 (3.35) | T test p=0.967 | 12.4 (3.31) |
| Median [Q1, Q3] | 10.0 [10.0, 16.0] | 12.5 [10.0, 16.0] | KW test p=0.867 | 10.0 [10.0, 16.0] |
| **CHANGE IN FI02** |  |  |  |  |
| Mean (SD) | 76.6 (7.62) | 76.5 (7.70) | T test p=0.931 | 76.5 (7.63) |
| Median [Q1, Q3] | 80.0 [80.0, 80.0] | 80.0 [80.0, 80.0] | KW test p=0.931 | 80.0 [80.0, 80.0] |
| **PFchange_day0** |  |  |  |  |
| Mean (SD) | 18.3 (137) | 21.6 (58.6) | T test p=0.884 | 20.3 (96.1) |
| Median [Q1, Q3] | 16.0 [-5.00, 42.3] | 6.00 [-6.88, 34.5] | KW test p=0.278 | 8.00 [-5.02, 39.0] |
| Missing | 2 (4.9%) | 0 (0%) |  | 2 (1.9%) |
| **PFchange_day1** |  |  |  |  |
| Mean (SD) | 30.5 (140) | 35.8 (74.8) | T test p=0.83 | 33.7 (105) |
| Median [Q1, Q3] | 44.0 [-6.50, 94.3] | 18.0 [-8.64, 56.5] | KW test p=0.257 | 25.5 [-8.16, 75.0] |
| Missing | 2 (4.9%) | 5 (8.1%) |  | 7 (6.8%) |
| **PFchange_day2** |  |  |  |  |
| Mean (SD) | 39.2 (149) | 33.7 (64.7) | T test p=0.843 | 35.9 (106) |
| Median [Q1, Q3] | 42.8 [19.7, 99.1] | 10.8 [-4.61, 64.5] | KW test p=0.0531 | 27.6 [-1.69, 83.4] |
| Missing | 8 (19.5%) | 13 (21.0%) |  | 21 (20.4%) |
| **PFchange_day3** |  |  |  |  |
| Mean (SD) | 35.5 (154) | 29.1 (58.1) | T test p=0.829 | 31.8 (109) |
| Median [Q1, Q3] | 59.5 [11.8, 90.9] | 12.0 [-9.00, 49.3] | KW test p=0.0157 | 40.5 [-0.500, 72.9] |
| Missing | 11 (26.8%) | 21 (33.9%) |  | 32 (31.1%) |
| **Pa02_pre** |  |  |  |  |
| Mean (SD) | 76.2 (3.04) | 76.1 (3.08) | T test p=0.946 | 76.1 (3.05) |
| Median [Q1, Q3] | 78.0 [76.0, 78.0] | 77.0 [76.0, 78.0] | KW test p=0.997 | 78.0 [76.0, 78.0] |
| **Pa02_post** |  |  |  |  |
| Mean (SD) | 165 (11.9) | 165 (12.1) | T test p=0.932 | 165 (12.0) |
| Median [Q1, Q3] | 171 [168, 171] | 170 [168, 171] | KW test p=0.997 | 171 [168, 171] |
| **Fi02_pre** |  |  |  |  |
| Mean (SD) | 82.6 (5.71) | 82.8 (6.05) | T test p=0.825 | 82.7 (5.89) |
| Median [Q1, Q3] | 80.0 [80.0, 90.0] | 80.0 [80.0, 90.0] | KW test p=0.91 | 80.0 [80.0, 90.0] |
| **Fi02_post** |  |  |  |  |
| Mean (SD) | 159 (10.4) | 159 (10.8) | T test p=0.952 | 159 (10.6) |
| Median [Q1, Q3] | 160 [155, 170] | 160 [155, 170] | KW test p=0.961 | 160 [155, 170] |
| **Pa02:Fi02_pre** |  |  |  |  |
| Mean (SD) | 0.926 (0.0576) | 0.923 (0.0598) | T test p=0.798 | 0.924 (0.0587) |
| Median [Q1, Q3] | 0.878 [0.875, 0.975] | 0.878 [0.875, 0.975] | KW test p=0.813 | 0.878 [0.875, 0.975] |
| **Pa02:Fi02_post** |  |  |  |  |
| Mean (SD) | 1.04 (0.0347) | 1.03 (0.0354) | T test p=0.774 | 1.03 (0.0350) |
| Median [Q1, Q3] | 1.01 [1.01, 1.07] | 1.01 [1.01, 1.07] | KW test p=0.851 | 1.01 [1.01, 1.07] |
| **PEEP_pre** |  |  |  |  |
| Mean (SD) | 12.4 (3.41) | 12.1 (3.35) | T test p=0.668 | 12.2 (3.36) |
| Median [Q1, Q3] | 10.0 [10.0, 16.0] | 10.0 [10.0, 16.0] | KW test p=0.673 | 10.0 [10.0, 16.0] |
| **PEEP_post** |  |  |  |  |
| Mean (SD) | 24.8 (3.26) | 24.5 (3.50) | T test p=0.636 | 24.6 (3.39) |
| Median [Q1, Q3] | 26.0 [25.0, 26.0] | 26.0 [25.0, 26.0] | KW test p=0.653 | 26.0 [25.0, 26.0] |
| **Change_in_Pa02:Fi02** |  |  |  |  |
| Mean (SD) | 0.109 (0.0248) | 0.110 (0.0263) | T test p=0.845 | 0.110 (0.0256) |
| Median [Q1, Q3] | 0.118 [0.0938, 0.128] | 0.118 [0.0938, 0.128] | KW test p=0.777 | 0.118 [0.0938, 0.128] |
| **%Change_in_Pa02** |  |  |  |  |
| Mean (SD) | 1.16 (0.0818) | 1.16 (0.0827) | T test p=0.922 | 1.16 (0.0819) |
| Median [Q1, Q3] | 1.19 [1.16, 1.21] | 1.21 [1.16, 1.21] | KW test p=1 | 1.21 [1.16, 1.21] |
| **%Change_in_Fi02** |  |  |  |  |
| Mean (SD) | 0.931 (0.105) | 0.927 (0.108) | T test p=0.847 | 0.928 (0.106) |
| Median [Q1, Q3] | 0.889 [0.889, 1.00] | 0.889 [0.889, 1.00] | KW test p=0.866 | 0.889 [0.889, 1.00] |
| **%Change_in_Pa02:Fi02** |  |  |  |  |
| Mean (SD) | 0.120 (0.0335) | 0.122 (0.0352) | T test p=0.808 | 0.121 (0.0344) |
| Median [Q1, Q3] | 0.135 [0.0962, 0.146] | 0.135 [0.0962, 0.146] | KW test p=0.777 | 0.135 [0.0962, 0.146] |
| **%Change_in_PEEP** |  |  |  |  |
| Mean (SD) | 1.10 (0.465) | 1.12 (0.456) | T test p=0.864 | 1.11 (0.457) |
| Median [Q1, Q3] | 0.800 [0.625, 1.60] | 1.15 [0.625, 1.60] | KW test p=0.901 | 0.800 [0.625, 1.60] |

| Descriptive Summary by Treat | | | |
| --- | --- | --- | --- |
|  | **NO (N=41)** | **EPO (N=62)** | **Total (N=103)** |
| **%Change_in_Pa02_10** |  |  |  |
| Yes | 41 (100%) | 62 (100%) | 103 (100%) |
| No | 0 (0%) | 0 (0%) | 0 (0%) |
| **%Change_in_Fi02_10** |  |  |  |
| Yes | 41 (100%) | 62 (100%) | 103 (100%) |
| No | 0 (0%) | 0 (0%) | 0 (0%) |
| **%Change_in_Pa02:Fi02_10** |  |  |  |
| Yes | 27 (65.9%) | 42 (67.7%) | 69 (67.0%) |
| No | 14 (34.1%) | 20 (32.3%) | 34 (33.0%) |
| **%Change_in_PEEP_10** |  |  |  |
| Yes | 41 (100%) | 62 (100%) | 103 (100%) |
| No | 0 (0%) | 0 (0%) | 0 (0%) |

| Descriptive Summary by Treat | | | | |
| --- | --- | --- | --- | --- |
|  | **NO (N=41)** | **EPO (N=62)** | **Chi-square test p-value** | **Total (N=103)** |
| **Sex** |  |  |  |  |
| F | 16 (39.0%) | 24 (38.7%) |  | 40 (38.8%) |
| M | 25 (61.0%) | 38 (61.3%) | Chi-sq p=1 /Fisher p=1 | 63 (61.2%) |
| **ETHNICITY** |  |  |  |  |
| A | 2 (4.9%) | 3 (4.8%) |  | 5 (4.9%) |
| B | 10 (24.4%) | 8 (12.9%) | Chi-sq p=0.53 /Fisher p=0.53(^f) | 18 (17.5%) |
| H | 14 (34.1%) | 20 (32.3%) |  | 34 (33.0%) |
| W | 10 (24.4%) | 8 (12.9%) |  | 27 (26.2%) |
| Missing | 6 (14.6%) | 13 (21.0%) |  | 19 (18.4%) |
| **HTN** |  |  |  |  |
| N | 18 (43.9%) | 26 (41.9%) |  | 44 (42.7%) |
| Y | 23 (56.1%) | 35 (56.5%) | Chi-sq p=1 /Fisher p=1 | 58 (56.3%) |
| Missing | 0 (0%) | 1 (1.6%) |  | 1 (1.0%) |
| **CAD** |  |  |  |  |
| N | 35 (85.4%) | 52 (83.9%) |  | 87 (84.5%) |
| Y | 6 (14.6%) | 9 (14.5%) | Chi-sq p=1 /Fisher p=1 | 15 (14.6%) |
| Missing | 0 (0%) | 1 (1.6%) |  | 1 (1.0%) |
| **NIDDM** |  |  |  |  |
| N | 33 (80.5%) | 43 (69.4%) |  | 76 (73.8%) |
| Y | 8 (19.5%) | 18 (29.0%) | Chi-sq p=0.366 /Fisher p=0.355 | 26 (25.2%) |
| Missing | 0 (0%) | 1 (1.6%) |  | 1 (1.0%) |
| **IDDM** |  |  |  |  |
| N | 35 (85.4%) | 53 (85.5%) |  | 88 (85.4%) |
| Y | 6 (14.6%) | 8 (12.9%) | Chi-sq p=1 /Fisher p=1 | 14 (13.6%) |
| Missing | 0 (0%) | 1 (1.6%) |  | 1 (1.0%) |
| **PAH** |  |  |  |  |
| N | 39 (95.1%) | 61 (98.4%) |  | 100 (97.1%) |
| Y | 2 (4.9%) | 0 (0%) | Chi-sq p=0.311 /Fisher p=0.159(^f) | 2 (1.9%) |
| Missing | 0 (0%) | 1 (1.6%) |  | 1 (1.0%) |
| **CHF** |  |  |  |  |
| N | 38 (92.7%) | 61 (98.4%) |  | 99 (96.1%) |
| Y | 3 (7.3%) | 0 (0%) | Chi-sq p=0.122 /Fisher p=0.0621(^f) | 3 (2.9%) |
| Missing | 0 (0%) | 1 (1.6%) |  | 1 (1.0%) |
| **Current Smoker/Vap** |  |  |  |  |
| N | 40 (97.6%) | 59 (95.2%) |  | 99 (96.1%) |
| Y | 1 (2.4%) | 2 (3.2%) | Chi-sq p=1 /Fisher p=1(^f) | 3 (2.9%) |
| Missing | 0 (0%) | 1 (1.6%) |  | 1 (1.0%) |
| **COPD** |  |  |  |  |
| N | 36 (87.8%) | 57 (91.9%) |  | 93 (90.3%) |
| Y | 5 (12.2%) | 4 (6.5%) | Chi-sq p=0.53 /Fisher p=0.479(^f) | 9 (8.7%) |
| Missing | 0 (0%) | 1 (1.6%) |  | 1 (1.0%) |
| **OSA** |  |  |  |  |
| N | 38 (92.7%) | 55 (88.7%) |  | 93 (90.3%) |
| Y | 3 (7.3%) | 7 (11.3%) | Chi-sq p=0.744 /Fisher p=0.736(^f) | 10 (9.7%) |
| **Asthma** |  |  |  |  |
| N | 35 (85.4%) | 52 (83.9%) |  | 87 (84.5%) |
| Y | 6 (14.6%) | 9 (14.5%) | Chi-sq p=1 /Fisher p=1 | 15 (14.6%) |
| Missing | 0 (0%) | 1 (1.6%) |  | 1 (1.0%) |
| **Past or current Cancer** |  |  |  |  |
| N | 38 (92.7%) | 51 (82.3%) |  | 89 (86.4%) |
| Y | 3 (7.3%) | 10 (16.1%) | Chi-sq p=0.296 /Fisher p=0.233 | 13 (12.6%) |
| Missing | 0 (0%) | 1 (1.6%) |  | 1 (1.0%) |
| **Immunocomrpomised** |  |  |  |  |
| N | 40 (97.6%) | 54 (87.1%) |  | 94 (91.3%) |
| Y | 1 (2.4%) | 7 (11.3%) | Chi-sq p=0.197 /Fisher p=0.139(^f) | 8 (7.8%) |
| Missing | 0 (0%) | 1 (1.6%) |  | 1 (1.0%) |
| **Fentanyl gtt** |  |  |  |  |
| N | 19 (46.3%) | 40 (64.5%) |  | 59 (57.3%) |
| Y | 22 (53.7%) | 22 (35.5%) | Chi-sq p=0.105 /Fisher p=0.103 | 44 (42.7%) |
| **Hydromorphone gtt** |  |  |  |  |
| N | 10 (24.4%) | 10 (16.1%) |  | 20 (19.4%) |
| Y | 31 (75.6%) | 51 (82.3%) | Chi-sq p=0.457 /Fisher p=0.324 | 82 (79.6%) |
| Missing | 0 (0%) | 1 (1.6%) |  | 1 (1.0%) |
| **Morphine gtt** |  |  |  |  |
| N | 40 (97.6%) | 55 (88.7%) |  | 95 (92.2%) |
| Y | 1 (2.4%) | 7 (11.3%) | Chi-sq p=0.205 /Fisher p=0.141(^f) | 8 (7.8%) |
| **Ketamine gtt** |  |  |  |  |
| N | 36 (87.8%) | 53 (85.5%) |  | 89 (86.4%) |
| Y | 5 (12.2%) | 9 (14.5%) | Chi-sq p=0.966 /Fisher p=1 | 14 (13.6%) |
| **Midazolam gtt** |  |  |  |  |
| N | 5 (12.2%) | 41 (66.1%) |  | 46 (44.7%) |
| Y | 36 (87.8%) | 21 (33.9%) | Chi-sq p=<0.001 /Fisher p=<0.001 | 57 (55.3%) |
| **Precedex gtt** |  |  |  |  |
| N | 27 (65.9%) | 18 (29.0%) |  | 45 (43.7%) |
| Y | 14 (34.1%) | 44 (71.0%) | Chi-sq p=<0.001 /Fisher p=<0.001 | 58 (56.3%) |
| **Propofol gtt** |  |  |  |  |
| N | 9 (22.0%) | 8 (12.9%) |  | 17 (16.5%) |
| Y | 32 (78.0%) | 54 (87.1%) | Chi-sq p=0.347 /Fisher p=0.281 | 86 (83.5%) |
| **Ciastracurium/VEC gtt** |  |  |  |  |
| N | 5 (12.2%) | 4 (6.5%) |  | 9 (8.7%) |
| Y | 36 (87.8%) | 58 (93.5%) | Chi-sq p=0.513 /Fisher p=0.478(^f) | 94 (91.3%) |
| **Steroids** |  |  |  |  |
| N | 3 (7.3%) | 1 (1.6%) |  | 4 (3.9%) |
| Y | 38 (92.7%) | 61 (98.4%) | Chi-sq p=0.344 /Fisher p=0.299(^f) | 99 (96.1%) |
| **Tocilizumab/ANTI IL6** |  |  |  |  |
| N | 26 (63.4%) | 41 (66.1%) |  | 67 (65.0%) |
| Y | 15 (36.6%) | 21 (33.9%) | Chi-sq p=0.943 /Fisher p=0.834 | 36 (35.0%) |
| **Remdesivir** |  |  |  |  |
| N | 34 (82.9%) | 55 (88.7%) |  | 89 (86.4%) |
| Y | 7 (17.1%) | 7 (11.3%) | Chi-sq p=0.586 /Fisher p=0.558 | 14 (13.6%) |
| **Hydroxychloroquine** |  |  |  |  |
| N | 5 (12.2%) | 7 (11.3%) |  | 12 (11.7%) |
| Y | 36 (87.8%) | 55 (88.7%) | Chi-sq p=1 /Fisher p=1(^f) | 91 (88.3%) |
| **Azithromycin** |  |  |  |  |
| N | 3 (7.3%) | 3 (4.8%) |  | 6 (5.8%) |
| Y | 38 (92.7%) | 59 (95.2%) | Chi-sq p=0.924 /Fisher p=0.68(^f) | 97 (94.2%) |
| **Other Abx** |  |  |  |  |
| Y | 41 (100%) | 61 (98.4%) |  | 102 (99.0%) |
| N | 0 (0%) | 1 (1.6%) | Chi-sq p=1 /Fisher p=1(^f) | 1 (1.0%) |
| **Antifungals** |  |  |  |  |
| N | 35 (85.4%) | 51 (82.3%) |  | 86 (83.5%) |
| Y | 5 (12.2%) | 11 (17.7%) | Chi-sq p=0.666 /Fisher p=0.583 | 16 (15.5%) |
| Missing | 1 (2.4%) | 0 (0%) |  | 1 (1.0%) |
| **Prone Position** |  |  |  |  |
| N | 14 (34.1%) | 8 (12.9%) |  | 22 (21.4%) |
| Y | 27 (65.9%) | 54 (87.1%) | Chi-sq p=0.0198 /Fisher p=0.0139 | 81 (78.6%) |
| **Products Given** |  |  |  |  |
| N | 31 (75.6%) | 55 (88.7%) |  | 86 (83.5%) |
| Y | 10 (24.4%) | 7 (11.3%) | Chi-sq p=0.138 /Fisher p=0.105 | 17 (16.5%) |
| **On ECMO** |  |  |  |  |
| N | 39 (95.1%) | 61 (98.4%) |  | 100 (97.1%) |
| Y | 2 (4.9%) | 1 (1.6%) | Chi-sq p=0.714 /Fisher p=0.562(^f) | 3 (2.9%) |
| **Plasma Given** |  |  |  |  |
| N | 30 (73.2%) | 51 (82.3%) |  | 81 (78.6%) |
| Y | 11 (26.8%) | 11 (17.7%) | Chi-sq p=0.392 /Fisher p=0.329 | 22 (21.4%) |
| **Heparin gtt** |  |  |  |  |
| N | 18 (43.9%) | 14 (22.6%) |  | 32 (31.1%) |
| Y | 23 (56.1%) | 48 (77.4%) | Chi-sq p=0.0383 /Fisher p=0.0298 | 71 (68.9%) |
| **Systemic tPa** |  |  |  |  |
| N | 31 (75.6%) | 45 (72.6%) |  | 76 (73.8%) |
| Y | 10 (24.4%) | 17 (27.4%) | Chi-sq p=0.91 /Fisher p=0.821 | 27 (26.2%) |
| **Renal Dysfunction** |  |  |  |  |
| N | 12 (29.3%) | 21 (33.9%) |  | 33 (32.0%) |
| Y | 29 (70.7%) | 41 (66.1%) | Chi-sq p=0.784 /Fisher p=0.671 | 70 (68.0%) |
| **RRT** |  |  |  |  |
| N | 22 (53.7%) | 39 (62.9%) |  | 61 (59.2%) |
| Y | 19 (46.3%) | 23 (37.1%) | Chi-sq p=0.466 /Fisher p=0.414 | 42 (40.8%) |
| **Liver Dysfunction** |  |  |  |  |
| N | 25 (61.0%) | 44 (71.0%) |  | 69 (67.0%) |
| Y | 16 (39.0%) | 18 (29.0%) | Chi-sq p=0.4 /Fisher p=0.392 | 34 (33.0%) |
| **Initial Vent Settings** |  |  |  |  |
| AC | 14 (34.1%) | 40 (64.5%) |  | 54 (52.4%) |
| BIVENT | 1 (2.4%) | 0 (0%) | Chi-sq p=0.0119 /Fisher p=0.00379(^f) | 1 (1.0%) |
| PC | 1 (2.4%) | 0 (0%) |  | 1 (1.0%) |
| PRVC | 20 (48.8%) | 12 (19.4%) |  | 32 (31.1%) |
| PS | 4 (9.8%) | 7 (11.3%) |  | 11 (10.7%) |
| VC | 1 (2.4%) | 3 (4.8%) |  | 4 (3.9%) |
| **Immediate Vent Settings** |  |  |  |  |
| AC | 14 (34.1%) | 40 (64.5%) |  | 54 (52.4%) |
| BIVENT | 1 (2.4%) | 0 (0%) | Chi-sq p=0.0637 /Fisher p=0.0217(^f) | 1 (1.0%) |
| PC | 1 (2.4%) | 0 (0%) |  | 1 (1.0%) |
| PRVC | 17 (41.5%) | 14 (22.6%) |  | 31 (30.1%) |
| PS | 4 (9.8%) | 6 (9.7%) |  | 10 (9.7%) |
| PVC | 1 (2.4%) | 0 (0%) |  | 1 (1.0%) |
| VC | 1 (2.4%) | 2 (3.2%) |  | 3 (2.9%) |
| Missing | 2 (4.9%) | 0 (0%) |  | 2 (1.9%) |
| **Immediate Positive response** |  |  |  |  |
| N | 17 (41.5%) | 37 (59.7%) |  | 54 (52.4%) |
| Y | 22 (53.7%) | 25 (40.3%) | Chi-sq p=0.17 /Fisher p=0.152 | 47 (45.6%) |
| Missing | 2 (4.9%) | 0 (0%) |  | 2 (1.9%) |
| **Vent Settings, Day 1** |  |  |  |  |
| AC | 14 (34.1%) | 35 (56.5%) |  | 49 (47.6%) |
| DECEASED | 2 (4.9%) | 5 (8.1%) | Chi-sq p=0.0134 /Fisher p=0.00707(^f) | 7 (6.8%) |
| PRVC | 23 (56.1%) | 13 (21.0%) |  | 36 (35.0%) |
| PS | 2 (4.9%) | 6 (9.7%) |  | 8 (7.8%) |
| PC | 0 (0%) | 2 (3.2%) |  | 2 (1.9%) |
| VC | 0 (0%) | 1 (1.6%) |  | 1 (1.0%) |
| **Positive response, Day 1** |  |  |  |  |
| DECEASED | 2 (4.9%) | 5 (8.1%) |  | 7 (6.8%) |
| N | 10 (24.4%) | 29 (46.8%) | Chi-sq p=0.0378 /Fisher p=0.036(^f) | 39 (37.9%) |
| Y | 29 (70.7%) | 28 (45.2%) |  | 57 (55.3%) |
| **Vent Settings, Day 2** |  |  |  |  |
| AC | 12 (29.3%) | 34 (54.8%) |  | 46 (44.7%) |
| DECEASED | 5 (12.2%) | 13 (21.0%) | Chi-sq p=0.00382 /Fisher p=<0.001(^f) | 18 (17.5%) |
| NO ABG | 2 (4.9%) | 0 (0%) |  | 2 (1.9%) |
| PRVC | 19 (46.3%) | 8 (12.9%) |  | 27 (26.2%) |
| PS | 1 (2.4%) | 4 (6.5%) |  | 5 (4.9%) |
| Stand-by | 1 (2.4%) | 0 (0%) |  | 1 (1.0%) |
| APRV | 0 (0%) | 1 (1.6%) |  | 1 (1.0%) |
| SPONTANEOUS | 0 (0%) | 1 (1.6%) |  | 1 (1.0%) |
| VC | 0 (0%) | 1 (1.6%) |  | 1 (1.0%) |
| Missing | 1 (2.4%) | 0 (0%) |  | 1 (1.0%) |
| **Positive response, Day 2** |  |  |  |  |
| DECEASED | 5 (12.2%) | 12 (19.4%) |  | 17 (16.5%) |
| N | 9 (22.0%) | 25 (40.3%) | Chi-sq p=0.0589 /Fisher p=0.0418(^f) | 34 (33.0%) |
| NO ABG | 2 (4.9%) | 0 (0%) |  | 2 (1.9%) |
| Y | 24 (58.5%) | 24 (38.7%) |  | 48 (46.6%) |
| ADD FORMULA | 0 (0%) | 1 (1.6%) |  | 1 (1.0%) |
| Missing | 1 (2.4%) | 0 (0%) |  | 1 (1.0%) |
| **Vent Settings, Day 3** |  |  |  |  |
| AC | 11 (26.8%) | 25 (40.3%) |  | 36 (35.0%) |
| DECEASED | 8 (19.5%) | 21 (33.9%) | Chi-sq p=0.0717 /Fisher p=0.0277(^f) | 29 (28.2%) |
| NO ABG | 1 (2.4%) | 0 (0%) |  | 1 (1.0%) |
| PRVC | 16 (39.0%) | 9 (14.5%) |  | 25 (24.3%) |
| PS | 2 (4.9%) | 4 (6.5%) |  | 6 (5.8%) |
| Stand-by | 1 (2.4%) | 0 (0%) |  | 1 (1.0%) |
| APRV | 0 (0%) | 1 (1.6%) |  | 1 (1.0%) |
| SIMV | 0 (0%) | 1 (1.6%) |  | 1 (1.0%) |
| VC | 0 (0%) | 1 (1.6%) |  | 1 (1.0%) |
| Missing | 2 (4.9%) | 0 (0%) |  | 2 (1.9%) |
| **Positive response, Day 3** |  |  |  |  |
| DECEASED | 8 (19.5%) | 21 (33.9%) |  | 29 (28.2%) |
| N | 8 (19.5%) | 21 (33.9%) | Chi-sq p=0.0476 /Fisher p=0.0371(^f) | 29 (28.2%) |
| NO ABG | 1 (2.4%) | 0 (0%) |  | 1 (1.0%) |
| Y | 22 (53.7%) | 20 (32.3%) |  | 42 (40.8%) |
| Missing | 2 (4.9%) | 0 (0%) |  | 2 (1.9%) |
| **Rebound Hypoxemia** |  |  |  |  |
| N | 23 (56.1%) | 31 (50.0%) |  | 54 (52.4%) |
| Y | 18 (43.9%) | 31 (50.0%) | Chi-sq p=0.685 /Fisher p=0.554 | 49 (47.6%) |
| **VFD** |  |  |  |  |
| N | 36 (87.8%) | 59 (95.2%) |  | 95 (92.2%) |
| Y | 4 (9.8%) | 3 (4.8%) | Chi-sq p=0.545 /Fisher p=0.428(^f) | 7 (6.8%) |
| Missing | 1 (2.4%) | 0 (0%) |  | 1 (1.0%) |
| **30 day survival** |  |  |  |  |
| M | 1 (2.4%) | 0 (0%) |  | 1 (1.0%) |
| N | 28 (68.3%) | 54 (87.1%) | Chi-sq p=0.0495 /Fisher p=0.0293(^f) | 82 (79.6%) |
| Y | 12 (29.3%) | 8 (12.9%) |  | 20 (19.4%) |
| **Mortality** |  |  |  |  |
| N | 9 (22.0%) | 6 (9.7%) |  | 15 (14.6%) |
| Y | 32 (78.0%) | 56 (90.3%) | Chi-sq p=0.149 /Fisher p=0.0955 | 88 (85.4%) |
| **Terminal Extubation** |  |  |  |  |
| N | 23 (56.1%) | 39 (62.9%) |  | 62 (60.2%) |
| Y | 18 (43.9%) | 23 (37.1%) | Chi-sq p=0.628 /Fisher p=0.541 | 41 (39.8%) |
| **Expiried** |  |  |  |  |
| N | 12 (29.3%) | 5 (8.1%) |  | 17 (16.5%) |
| Y | 29 (70.7%) | 57 (91.9%) | Chi-sq p=0.0103 /Fisher p=0.00647 | 86 (83.5%) |

(^f) Fisher's exact p-value should be applied
